# Mapping of positive selection sites in the HIV genome in the context of RNA and protein structural constraints

**DOI:** 10.1186/1742-4690-8-S2-O42

**Published:** 2011-10-03

**Authors:** Joke Snoeck, Jacgues Fellay, Daniel C Douek, Amalio Telenti

**Affiliations:** 1 Institute of Microbiology, University Hospital Center and University of Lausanne, Lausanne, Switzerland; 2 Rega Institute for Medical Research, KU Leuven, Leuven, Belgium; 3 Global Health Institute, School of Life Sciences, EPFL, Lausanne, Switzerland; 4 Human Immunology Section, Vaccine Research Center, Nationa Institute of Allergy and Infectious Diseases, NIH, Bethesda, Maryland, USA

## Background

The HIV-1 genome is subject to great variability. On the one hand, there are recognized pressures that target the virus and resulting in escape and adaptation. On the other hand, there is a requirement for sequence conservation because of functional and structural constraints. Mapping the sites of positive selective pressure on the viral genome generates a reference for understanding the limits to viral escape, and can serve as a template for the discovery of sites of genetic conflict with known or unknown host proteins.

## Materials and methods

To build a thorough evolutionary, functional and structural map of the HIV-1 genome, full subtype B sequences were obtained from the Los Alamos database. We mapped sites under positive selective pressure (using the SLAC method in HYPHY), amino acid conservation, protein and RNA structure [1], overlapping coding frames, as well as CD8 T cell, CD4 T cell and antibody epitopes (based on the lists form the Los Alamos database website). Fisher exact test was used for univariate analysis, and either logistic regression or binary Firth's penalized-likelihood logistic regression was used in multivariate analysis. Statistical analyses were performed in R version 2.13.0 (http://www.r-project.org/).

## Results

Globally, 33% of amino acid positions were found to be variable and 12% of the genome was under positive selection. Because interrelated constraining and diversifying forces shape the viral genome, we included the variables from both classes of pressure in a multivariate model to predict conservation or positive selection: molecular structures (structured RNA and α-helix domains) predicted conservation while CD4 T cell and antibody epitopes were associated with positive selection. Gene-specific analyses showed instances of departure from the genome-wide estimates. For example, CD8 T cell epitopes were generally well conserved, except in *gp120*, where they were enriched for sites under positive selective pressure. Similarly, CD4 T cell epitopes were enriched for sites under positive selection genome-wide, but these epitopes were significantly more conserved in *gag.* Thus, the various constraints and selective pressures do not act evenly across the genome.

## Conclusions

Because the global map of the viral genome also identifies positive selected sites that are not in canonical CD8 T cell, CD4 T cell or antibody epitopes, it identifies a class of residues that may be targeted by other host selective pressures, such as innate immunity effectors. As an example, sequence adaptation has been observed in the viral capsid in rhesus macaques upon cross-species transmission of SIVsm, due to selective pressure provided by restrictive TRIMS alleles. In addition to informing the combined analysis of host and viral genetic information, the results of this study may therefore help reveal novel mechanisms of antiretroviral response.

## References

[B1] Watts JM Dang KK Gorelick RJ Leonard CW Bess JW Jr Swanstrom R Burch CL Weeks KM Architecture and secondary structure of an entire HIV-1 RNA genome Nature 2009 460 711 716 10.1038/nature08237 19661910 PMC2724670

